# Phenolic contents and antioxidant activities of Caucasian bee propolis at different locations, solvents, and temperatures

**DOI:** 10.1002/fsn3.4400

**Published:** 2024-08-11

**Authors:** Tuğba Nigar Bozkuş

**Affiliations:** ^1^ Laboratory Technology Program, Artvin Vocational School Artvin Çoruh University Artvin Turkey

**Keywords:** antioxidant activity, Caucasian bee, phenolic content, propolis

## Abstract

Propolis is a natural substance produced by honey bees by processing the secretions collected from various plants with their own enzymes. Caucasian honey bee is one of the productive bee races known in the world and its homeland in Türkiye is Northeastern Anatolia. This study is the first comprehensive research to determine, compare, and correlate the effects of various solvents and temperatures on 17 different Caucasian bee propolis obtained from different locations in terms of phenolic content and antioxidant activity. The highest total polyphenol and flavonoid contents (TPC and TFC) in Caucasian bee propolis extracts varied between 23.37–97.62 mg gallic acid per gram (GA/g) of propolis and 6.73–43.75 mg quercetin per gram (Q/g) of propolis in ethanolic extract at 60°C. While ferric reducing antioxidant power (FRAP) did not differ at both temperatures, the highest was determined in ethanolic extracts. Analyses of propolis samples according to locations did not show any significant differences in terms of TPC, TFC, and FRAP. By Pearson's correlation analysis, it was found that each of the extracts showed a positive correlation between TPC, TFC, and FRAP. As a result, it was revealed that the main difference in the phenolic content and antioxidant activities of propolis obtained from gene centers of Caucasian bee race was due to the polarity of the solvent used and temperature, and that there was no difference between locations within a single bee race. This pioneering study shows that Caucasian bee propolis has a high potential that can pave the way for different research in this field.

## Graphical abstract

This study is the first comprehensive research to determine, compare, and correlate the effects of various solvents and temperatures on 17 different Caucasian bee propolis obtained from different locations in terms of phenolic content and antioxidant activity. Results indicated that the main difference in the phenolic content and antioxidant activities of Caucasian bee propolis was due to the polarity of the solvent used and temperature, and there was no difference between the locations of a single bee race.

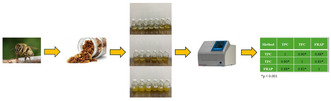

## 1 INTRODUCTION

Thanks to its diverse geography, ecology, and climate, Türkiye provides ideal conditions for beekeeping (Kahraman et al., 2010). Caucasian honey bee (*Apis mellifera caucasia*), known as one of the productive bee races of the world and among the notable honey bee races in Anatolia, is renowned for its long tongue, prolific use of propolis, low swarming tendency, and substantial honey production (Gül & Nergiz, 2022; Uçak Koç & Karacaoğlu, 2011). These bees originate from Northeastern Anatolia, thriving in the temperate climate and high‐altitude areas of this region (Akyol et al., 2014). Artvin, one of the key provinces in Türkiye for queen bee and honey production, serves as a crucial gene center, preserving and fostering the Caucasian bee race in the beekeeping industry (Gül & Nergiz, 2022).

Propolis is a rich and scented natural product, collected from various plant sources, which is used to protect honey bees from entrances, from external influences, and to seal holes in honeycombs (Orsatti et al., 2010; Wagh, 2013). Propolis contains over 300 chemical components, including polyphenols (flavonoid aglycones, phenol acids and ester, phenol aldehydes, alcohol, and ketones), coumarins, sesquiterpene quinones, steroids, and inorganic compounds (Lotfy, 2006).

The chemical contents of propolis may vary based on the factors, such as its geographic location, vegetative source, climate, collection time, or the genus of honey bees (Gong et al., 2012; Moreno et al., 2000; Nagai et al., 2003). Because of the biodiversity of the plants of each bee's visiting area, the chemical composition is very variable and complex (Liberio et al., 2009).

The plant source and aggregation site are also of great importance in the biological activity of propolis. Studies have shown that the presence of flavonoids and polyphenol compounds is responsible for the biological and pharmacological activity of many propolis, especially the antioxidant activity (Kanbur et al., 2009).

Since it is difficult to use propolis in its natural solid state, it is necessary to purify with solvent extraction. In this process, polyphenolic parts are preserved by removing inert substances (Pietta et al., 2002). The content of propolis can vary depending on the solvent used while preparing the extract (Nagai et al., 2003). Since different solvents will dissolve and extract different components in propolis, propolis extraction methods can affect the activity of propolis (Sforcin, 2007). Therefore, in this study, water, which is easily found in nature and the most suitable solvent for use, phosphate‐buffered saline (PBS) as it mimics the ion concentration, osmolarity, and pH of body fluids, and as used in many studies, ethanol that is stated to be one of the solvents in which propolis dissolves best, were used as solvents in propolis extraction.

Although there are different studies on the Caucasian bee race in the literature (Adl et al., 2007; Akyol et al., 2014; Ceylan et al., 2019; Uçak Koç & Karacaoğlu, 2011), there is no study on determining the phenolic contents, antioxidant activities, and correlations of various propolis samples obtained from the own gene region of this bee race. Therefore, in this study, it was aimed to determine the phenolic contents and antioxidant activities of the aqueous, PBS, and ethanolic extracts of Caucasian bee propolis samples obtained from 17 different locations of Artvin in Türkiye at different temperatures (37 and 60°C) and the correlation between them.

## 2 MATERIALS AND METHODS

### 2.1 Propolis samples

Caucasian bee natural propolis samples were supplied by Artvin Beekeepers Association from 17 different locations of Artvin region, which is one of the gene centers of Caucasian bees in Türkiye. These samples were powdered and stored in the refrigerator at −20°C. Then, each of the propolis samples was numbered from 1 to 17 according to the location where they were taken. The numbering is as follows: (1) Artvin‐Central, (2) Artvin‐Alabalık Village, (3) Artvin‐Kafkasör Plateau, (4) Artvin‐Seyitler Village, (5) Artvin‐Varlık Village, (6) Artvin‐Varyant, (7) Ardanuç‐Aydın Village, (8) Ardanuç‐Müezzinler Village, (9) Arhavi‐Central, (10) Borçka‐Karşıköy, (11) Hatila Valley‐Taşlıca Village, (12) Hopa‐Central, (13) Kemalpaşa‐Köprücük Village, (14) Murgul‐Kabaca Village, (15) Şavşat‐Çoraklı Village, (16) Şavşat‐Dereiçi Village, and (17) Yusufeli‐Central.

### 2.2 Preparation of Caucasian bee propolis extracts

The powdered Caucasian bee propolis samples were weighed on a sensitive balance to achieve a weight of 0.625 g and then transferred into reagent bottles with a volume of 50 mL each. Propolis samples were prepared with three different solvents. Distilled water, PBS (pH 7.4), and absolute ethanol with a volume of 25 mL each were added to separate reagent bottles and vortexed. They were incubated in a shaker incubator at 37 and 60°C at 150 rpm for 24 h with continuous shaking to dissolve propolis samples. After 24 h of incubation, propolis extracts were centrifuged at 2057 *g* for 10 min and filtered with the help of a filter paper. Thus, 25 mg/mL stock propolis extracts with aqueous, PBS, and ethanol at 37 and 60°C were prepared and stored in the refrigerator at +4°C in the dark to be used in the necessary experiments.

### 2.3 Determination of total polyphenol content (TPC)

The total polyphenol content in the extracts was quantified using a spectrophotometric method based on a modified version of the Folin–Ciocalteu technique. This method relies on the reduction of phosphotungstic acid [H_3_P(W_3_O_10_)_4_] to phosphotungstic blue in an alkaline medium, where the resulting absorbance is directly proportional to the concentration of aromatic phenolic groups (Horzic et al., 2009). In this procedure, 12.5 μL of propolis extract, 62.5 μL of a 1:10 diluted Folin–Ciocalteu reagent, and 125 μL of a 20% sodium carbonate (Na_2_CO_3_) solution were added to 96‐well microplates. The mixture was then incubated for 30 min at room temperature in the dark. Following incubation, absorbance was measured at 700 nm using a microplate reader (Thermo Scientific™ Multiskan™ SkyHigh Microplate Spectrophotometer). A calibration curve was established using gallic acid (GA) as the standard. The total polyphenol content was expressed as mg of gallic acid equivalents (GAE) per gram of propolis.

### 2.4 Determination of total flavonoid content (TFC)

The total flavonoid content of the extracts was determined using the aluminum chloride (AlCl_3_) colorimetric method. This method is based on the formation of acid‐stable complexes between AlCl_3_ and flavones or flavonols that contain C‐4 keto groups and C‐3 or C‐5 hydroxyl groups. Additionally, AlCl_3_ can form complexes with the ortho‐dihydroxyl groups present in the A‐ or B‐rings of flavonoids (Chang et al., 2002). In the procedure, 20 μL of propolis extract, 172 μL of 80% ethanol, 4 μL of 10% aluminum chloride (AlCl_3_), and 4 μL of 1 M potassium acetate (KCH_3_COO) solution were added to 96‐well microplates. The mixture was incubated for 40 min at room temperature in the dark. After the incubation period, absorbance was measured at 415 nm using a microplate reader. Quercetin was used to create the calibration curve, and the total flavonoid content was expressed as mg of quercetin equivalents (QE) per gram of propolis.

### 2.5 Determination of ferric (Fe^3+^) reducing antioxidant power (FRAP)

The reducing power of the extracts was assessed using the ferricyanide method (Mohtar et al., 2020), with modifications based on the Oyaizu method (1986). In this approach, 40 μL of propolis extract, 100 μL of 0.2 M phosphate buffer (pH 6.6), and 100 μL of potassium hexacyanoferrate [K_3_Fe(CN)_6_] were mixed and incubated at 50°C for 20 min in the dark. Following incubation, the mixture was cooled, and 100 μL of 10% trichloroacetic acid (TCA) was added. The resulting mixture was centrifuged at 3000 *g* for 10 min. From the upper phase of the centrifuged samples, 100 μL was transferred to a 96‐well microplate, and 100 μL of distilled water along with 20 μL of iron (III) chloride (FeCl_3_) was added. The final mixture was incubated for 5 min at room temperature in the dark. Absorbance was then measured at 700 nm using a microplate reader. Trolox was employed as a standard to construct the calibration curve, and the antioxidant potential of the propolis was expressed as mg Trolox equivalents (TE) per gram of propolis.

### 2.6 Statistical analysis

The obtained values were expressed as the arithmetic mean ± standard deviation (*x* ± SD). Data were statistically evaluated using the R Program (Version 4.1). To reveal the relationship between the groups, normality analysis was performed, and it was seen that the data were not normally distributed. The data were evaluated with Kruskal–Wallis analysis and Paired Comparative Post Hoc Test analyses were used between the groups that were significant according to the results of this analysis. Comparisons were made between 17 propolis extracts in terms of total polyphenol content, total flavonoid content, and ferric (Fe^3+^) reducing antioxidant power, and those with *p* < .05 were considered significant. In addition, Pearson's correlation test was used to calculate the correlation between the antioxidant activity (FRAP) with TPC and TFC using the R Program, Version 4.1.

## 3 RESULTS

The 17 propolis samples did not differ statistically in terms of polyphenol content (*χ* ^2^ = 13.25, *p* = .625), but they differed in terms of solvent (*χ* ^2^ = 67.46, *p* < .001). As a result of the Double Comparative Post Hoc Test, the reason for the difference was that the propolis samples were significantly better dissolved in ethanol than in water and PBS. The 17 propolis samples differed statistically in terms of temperature (*χ* ^2^ = 6.76, *p* < .05). As a result of the Double Comparative Post Hoc Test, the reason for the difference was that the propolis samples dissolved significantly better at 60°C. The results of the total polyphenol content determination of the extracts are given in Table 1.

**TABLE 1:** The total polyphenol content of Caucasian bee propolis extracts (arithmetic mean ± SD, *n* = 4) (mg GA/g propolis).

Propolis samples	Aqueous extract (37°C)	PBS (37°C)	Ethanolic (37°C)	Aqueous extract (60°C)	PBS (60°C)	Ethanolic extract (60°C)
1	8.48 ± 0.07	10.00 ± 0.28	55.83 ± 0.86	14.32 ± 0.34	14.90 ± 0.18	63.15 ± 1.35
2	10.50 ± 0.26	8.83 ± 0.13	29.40 ± 1.74	14.39 ± 0.76	13.50 ± 0.20	34.67 ± 1.10
3	6.65 ± 0.26	9.36 ± 0.31	58.40 ± 0.70	14.24 ± 0.73	10.04 ± 0.56	73.45 ± 1.79
4	9.73 ± 0.24	10.35 ± 0.35	51.43 ± 1.65	14.75 ± 0.67	13.87 ± 0.31	53.35 ± 1.06
5	5.82 ± 0.47	9.04 ± 0.69	78.53 ± 3.81	10.24 ± 0.86	12.06 ± 0.40	82.87 ± 6.72
6	5.94 ± 0.25	7.28 ± 0.12	31.17 ± 3.18	6.94 ± 0.19	10.47 ± 0.53	41.01 ± 0.86
7	11.18 ± 0.22	15.61 ± 0.52	80.43 ± 0.93	28.43 ± 1.17	26.65 ± 1.28	97.62 ± 3.00
8	6.16 ± 0.34	10.89 ± 0.26	64.43 ± 3.59	7.87 ± 0.78	12.04 ± 0.41	68.57 ± 3.96
9	7.37 ± 0.14	6.75 ± 0.17	22.85 ± 2.65	9.56 ± 0.24	8.43 ± 0.17	23.37 ± 2.40
10	7.96 ± 0.20	8.04 ± 0.37	25.43 ± 0.43	10.84 ± 0.18	11.06 ± 0.31	32.79 ± 1.85
11	8.00 ± 0.35	9.34 ± 0.22	29.97 ± 1.42	14.11 ± 0.32	16.27 ± 0.65	37.05 ± 1.46
12	10.15 ± 0.20	9.73 ± 0.33	31.97 ± 1.68	15.49 ± 0.14	11.63 ± 0.15	30.03 ± 1.53
13	15.38 ± 0.15	14.38 ± 0.29	53.80 ± 2.83	19.96 ± 1.00	18.43 ± 1.06	53.43 ± 1.15
14	5.73 ± 0.13	7.16 ± 0.08	44.17 ± 1.19	10.58 ± 0.52	11.29 ± 0.41	43.92 ± 0.57
15	7.18 ± 0.44	8.05 ± 0.28	26.63 ± 0.72	7.66 ± 0.20	13.80 ± 0.24	31.09 ± 1.56
16	8.05 ± 0.27	11.11 ± 0.23	72.37 ± 4.82	8.34 ± 0.52	13.61 ± 0.14	87.92 ± 6.14
17	9.59 ± 0.19	8.47 ± 0.89	35.07 ± 0.40	10.75 ± 0.20	10.43 ± 0.30	35.52 ± 2.30

The 17 propolis samples did not differ statistically in terms of flavonoid content (*χ* ^2^ = 7.76, *p* = .956), but they differed in terms of solvent (*χ* ^2^ = 69.29, *p* < .001). As a result of the Double Comparative Post Hoc Test, the reason for the difference was that the propolis samples were significantly better dissolved in ethanol than in water and PBS. The 17 propolis samples differed statistically in terms of temperature (*χ* ^2^ = 5.39, *p* < .05). As a result of the Double Comparative Post Hoc Test, the reason for the difference was that the propolis samples dissolved significantly better at 60°C. The results of the total flavonoid content determination of the extracts are given in Table 2.

**TABLE 2:** Total flavonoid content of Caucasian bee propolis extracts (arithmetic mean ± SD, *n* = 4) (mg Q/g propolis).

Propolis samples	Aqueous extract (37°C)	PBS (37°C)	Ethanolic extract (37°C)	Aqueous extract (60°C)	PBS (60°C)	Ethanolic extract (60°C)
1	0.46 ± 0.02	0.74 ± 0.09	22.49 ± 1.29	0.89 ± 0.08	2.35 ± 0.16	20.70 ± 1.93
2	0.81 ± 0.30	0.98 ± 0.11	7.18 ± 0.08	0.74 ± 0.06	1.07 ± 0.03	8.41 ± 1.14
3	0.21 ± 0.02	0.45 ± 0.10	14.65 ± 0.31	0.79 ± 0.07	0.65 ± 0.07	14.48 ± 0.68
4	0.67 ± 0.04	1.02 ± 0.13	18.86 ± 0.08	1.09 ± 0.11	1.14 ± 0.05	18.18 ± 1.61
5	0.37 ± 0.02	0.76 ± 0.18	34.32 ± 1.49	0.80 ± 0.07	1.44 ± 0.16	30.21 ± 0.49
6	1.61 ± 0.16	4.03 ± 0.55	7.78 ± 0.67	3.39 ± 0.35	3.93 ± 1.44	6.73 ± 1.69
7	0.68 ± 0.11	0.72 ± 0.09	28.42 ± 1.08	1.78 ± 0.12	2.04 ± 0.08	26.00 ± 2.27
8	0.59 ± 0.01	0.61 ± 0.06	41.73 ± 1.44	1.01 ± 0.09	1.23 ± 0.25	41.98 ± 4.45
9	0.54 ± 0.04	0.84 ± 0.15	7.64 ± 0.27	0.77 ± 0.05	0.66 ± 0.01	7.61 ± 0.43
10	0.55 ± 0.01	0.79 ± 0.12	7.37 ± 0.12	1.34 ± 0.06	2.12 ± 0.39	7.78 ± 0.36
11	0.51 ± 0.10	0.95 ± 0.14	6.48 ± 0.33	1.27 ± 0.38	2.51 ± 0.32	7.12 ± 0.45
12	0.96 ± 0.06	1.05 ± 0.07	6.38 ± 0.10	1.35 ± 0.05	0.91 ± 0.04	7.29 ± 0.24
13	0.70 ± 0.12	1.00 ± 0.12	11.59 ± 0.54	1.08 ± 0.04	1.58 ± 0.15	13.31 ± 1.45
14	0.40 ± 0.03	0.68 ± 0.19	12.15 ± 1.91	1.30 ± 0.31	1.99 ± 0.49	12.28 ± 0.45
15	2.16 ± 0.21	2.79 ± 0.17	7.05 ± 1.56	3.79 ± 0.28	4.88 ± 0.13	7.48 ± 0.91
16	0.67 ± 0.03	0.80 ± 0.19	45.17 ± 2.00	1.05 ± 0.06	2.21 ± 0.48	43.75 ± 1.74
17	1.08 ± 0.14	1.78 ± 0.14	14.03 ± 2.90	1.42 ± 0.13	1.97 ± 0.24	9.19 ± 0.81

The 17 propolis samples did not differ statistically in terms of ferric (Fe^3+^) reducing antioxidant power (*χ* ^2^ = 20.43, *p* = .201), but they differed in terms of solvent (*χ* ^2^ = 62.64, *p* < .001). As a result of the Double Comparative Post Hoc Test, the reason for the difference was that the propolis samples were significantly better dissolved in ethanol than in water and PBS. The 17 propolis samples did not differ in terms of temperature (*χ* ^2^ = 2.68, *p* = .102). The results of the ferric (Fe^3+^) reducing antioxidant power determination of the extracts are given in Table 3.

**TABLE 3:** Ferric (Fe^3+^) reducing antioxidant power of Caucasian bee propolis extracts (arithmetic mean ± SD, *n* = 4) (mg T/g propolis).

Propolis samples	Aqueous extract (37°C)	PBS (37°C)	Ethanolic extract (37°C)	Aqueous extract (60°C)	PBS (60°C)	Ethanolic extract (60°C)
1	13.63 ± 0.27	17.10 ± 0.38	91.78 ± 1.43	10.78 ± 0.17	10.54 ± 0.11	59.29 ± 2.36
2	21.62 ± 0.39	18.92 ± 0.22	59.23 ± 1.95	27.85 ± 0.42	23.50 ± 0.38	60.41 ± 1.92
3	10.87 ± 0.39	15.77 ± 0.23	106.09 ± 0.08	12.40 ± 0.20	3.27 ± 0.07	62.50 ± 0.45
4	18.67 ± 0.47	25.08 ± 0.46	87.46 ± 1.82	28.12 ± 0.70	28.12 ± 0.36	91.96 ± 1.52
5	9.29 ± 0.32	20.97 ± 0.23	128.80 ± 2.47	10.74 ± 0.34	9.49 ± 0.30	79.38 ± 0.78
6	10.68 ± 0.32	13.29 ± 0.08	41.73 ± 0.45	6.29 ± 0.08	11.83 ± 0.23	24.91 ± 0.45
7	16.65 ± 0.18	24.71 ± 0.08	131.20 ± 2.72	19.46 ± 0.55	17.41 ± 0.35	77.67 ± 0.58
8	11.94 ± 0.17	19.82 ± 0.15	99.24 ± 1.43	7.89 ± 0.52	8.38 ± 0.35	58.06 ± 0.38
9	17.74 ± 0.18	19.12 ± 0.32	43.03 ± 0.32	20.54 ± 0.32	18.65 ± 0.44	43.58 ± 0.62
10	13.63 ± 0.66	14.62 ± 0.28	38.49 ± 0.98	8.30 ± 0.17	12.69 ± 0.33	27.79 ± 0.80
11	12.95 ± 2.05	15.00 ± 0.23	48.50 ± 0.71	6.86 ± 0.11	4.26 ± 0.38	26.38 ± 0.37
12	24.50 ± 0.15	22.00 ± 0.18	58.15 ± 0.49	29.88 ± 0.23	22.15 ± 0.44	58.35 ± 1.00
13	28.27 ± 0.15	25.85 ± 0.40	90.27 ± 0.62	35.19 ± 0.34	29.73 ± 0.78	91.08 ± 2.53
14	10.02 ± 0.45	13.97 ± 0.30	76.62 ± 0.68	11.24 ± 0.17	8.00 ± 0.45	56.83 ± 1.01
15	11.01 ± 0.64	13.57 ± 0.32	36.49 ± 0.89	12.83 ± 0.11	4.00 ± 0.34	19.92 ± 0.52
16	15.48 ± 0.26	23.27 ± 0.23	117.60 ± 1.47	10.23 ± 0.11	11.14 ± 0.47	76.29 ± 1.02
17	17.63 ± 0.15	16.40 ± 0.24	50.17 ± 0.35	7.69 ± 0.17	11.60 ± 0.70	28.41 ± 0.46

The Pearson correlation coefficient was used to correlate the antioxidant activities of the extracts with their polyphenol and flavonoid contents. With the correlation test, a significant correlation was found between TPC and TFC at a level of 0.90, between TPC and FRAP at a level of 0.88, and between TFC and FRAP at a level of 0.83 (Table 4).

**TABLE 4:** Pearson's correlation coefficient of antioxidant activity, total polyphenol and flavonoid contents of Caucasian bee propolis extracts.

Method	TPC	TFC	FRAP
TPC	1	.90*	.88*
TFC	.90*	1	.83*
FRAP	.88*	.83*	1

* Correlation is significant at *p* < .001.

## 4 DISCUSSION

In this study, propolis samples obtained from the regions that protect and host the Caucasian honey bee and taken from a total of 17 different locations were used. Phenolic content amounts and antioxidant activities of aqueous, PBS, and ethanolic extracts of Caucasian bee propolis samples and the correlation between them were determined. The 17 samples of Propolis did not differ in terms of polyphenol, flavonoids, and antioxidant activity. In addition, the ethanolic extracts of the samples of the Caucasian bee propolis showed higher polyphenol and flavonoid content and antioxidant activity than the PBS and aqueous extracts. As a result, it was decided that the Propolis samples would be best dissolved in ethanol. Since water and PBS did not have statistically significant differences, both solvents dissolved in a similar amount. In addition, as a result of the total polyphenol and flavonoid content analyses made in the extracts of propolis samples prepared at 37 and 60°C, the best dissolution was at 60°C, and since the lack of significant difference in the ferric (Fe^3+^) reducing antioxidant power analyses, the extracts dissolved at similar levels at both temperatures. According to Pearson's correlation analysis, the antioxidant activity, polyphenol and flavonoid contents of each of the extracts were positively correlated.

Propolis is a natural product containing high levels of phenols and displaying high antioxidant activities because it contains phenolic compounds (Kumazawa et al., 2004; Mohammadzadeh et al., 2007; Nagai et al., 2003). To date, although researchers have carried out studies on the phenolic content and antioxidant activity of propolis from various regions or countries (Alencar et al., 2007; Choi et al., 2006; Kalogeropoulos et al., 2009; Kumazawa et al., 2004; Mohammadzadeh et al., 2007; Moreira et al., 2008; Moreno et al., 2000; Silva et al., 2006), they have not focused on the bioactivities of bee races. In this regard, it has not been determined what kind of antioxidant properties propolis obtained from a particular bee race may have.

The Caucasian honey bee, known for its propolis collection and overuse feature, is one of the most common and prominent original honey bee races in Türkiye (Ceylan et al., 2019; Uçak Koç & Karacaoğlu, 2011). This study determined the phenolic content and antioxidant activity of propolis samples of a certain race and compared them with those of different solvents and temperatures. In this respect, this study is the first comprehensive study in this field. In addition, this study is important in terms of being a pioneer in various future research that can be done with bee products of Caucasian bee.

Propolis is a natural product produced by combining resin collected by honey bees from the flowers and sprouts of plants with beeswax and saliva enzymes (Bankova et al., 2002; Silici & Baysa, 2020). Due to the resin and wax‐like substances in its structure, raw propolis must be purified by extraction to reveal the biologically active components it contains (Pietta et al., 2002). The composition of propolis extracts depends on the type of raw propolis, extraction method and extraction solvent, and the phenolic composition varies depending on the polarity of the solvent used (Kolaylı & Birinci, 2024). Many studies have been conducted in the literature, in which the amounts of polyphenols, flavonoids, and antioxidants in all extracts were determined, based on samples obtained from different regions of the propolis extract with various solvents. However, it should be taken into consideration that each solvent can dissolve propolis at different ratios and therefore affect the activity of propolis and the results may be different (Bozkuş & Değer, 2022; Sforcin, 2007; Silva et al., 2012). Since propolis extracts prepared in water, PBS, and ethanol were used as solvents in this study, it is necessary to compare the results with those of extracts prepared with the same type of solvent.

In a study conducted by Bozkuş and Değer (2022), aqueous, ethanolic, glycerol, dimethyl sulfoxide (DMSO), and acetone extracts of propolis collected from various provinces in Türkiye were prepared for analysis. The total polyphenol content of these extracts ranged from 19.7 ± 0.29 to 141.2 ± 9.99 mg gallic acid per gram (GA/g) of propolis, the total flavonoid content ranged from 1.3 ± 0.12 to 55.3 ± 6.63 mg quercetin per gram (Q/g) of propolis, and the ferric (Fe^3+^) reducing antioxidant power ranged from 26.2 ± 8.57 to 273.8 ± 11.62 mg T/g (trolox per gram) of propolis. The study found that propolis dissolved most effectively in DMSO, followed by ethanol, acetone, glycerol, and water.

Comparatively, Kumazawa et al. (2004) found that the total polyphenol content of ethanolic extracts ranged from 99.5 ± 4.4 to 299 ± 0.5 mg GA/g propolis, and the total flavonoid content ranged from 2.5 ± 0.8 to 176 ± 1.7 mg Q/g propolis. Silva et al. (2012) examined hydro‐alcoholic, methanolic, and aqueous extracts of propolis from Portugal, reporting total phenolic content in aqueous extracts ranging from 18.52 ± 1.35 to 72.15 ± 1.20 mg GA/g propolis, and total flavonoid content ranging from 6.34 ± 0.55 to 42.30 ± 2.10 mg Q/g propolis.

Alencar et al. (2007) indicate that ethanolic extracts of Brazilian red propolis contained 232 ± 22.3 mg GA/g propolis of polyphenols and 43 ± 1.0 mg Q/g propolis of flavonoids. Frozza et al. (2013) reported that hydro‐alcoholic extracts of Brazilian red propolis had 151.55 ± 1.95 mg GA/g propolis of polyphenolic compounds as dry extract. Can et al. (2015) analyzed propolis from 15 locations in Azerbaijan, finding total phenolic content in ethanolic extracts ranging from 10.94 ± 0.15 to 79.23 ± 2.06 mg GA/g propolis. Ahn et al. (2007) found that the total polyphenol content in ethanolic extracts of Chinese propolis ranged from 42.9 ± 0.8 to 302 ± 4.3 mg GA/g propolis, with total flavonoid content ranging from 8.3 ± 3.7 to 188 ± 6.6 mg Q/g propolis.

The phenolic content and antioxidant activity of the ethanolic extract of Caucasian bee propolis samples were higher compared to those of other extracts from previous studies. Thus, while the results varied, they were still found to be consistent with those of other research. Kolaylı and Birinci (2024) found in their study that solvents with higher polarity contained more phenolic acid, while solvents with lower polarity exhibited higher amounts of flavonoids. The amount of phenolic substances varies due to the difference in solvent polarity, and therefore the antioxidant activity varies depending on the polyphenol and flavonoid contents (Kolaylı & Birinci, 2024). In addition, Kolaylı et al. (2023), in another study they conducted with Anatolian propolis obtained from seven different regions, stated that the balsam content or resin, which represents the extractable part in ethanol, is where the bioactive components of propolis are largely found. The aqueous extract demonstrated lower solubility than ethanol in this study, similar to findings in other studies. Consequently, the phenolic content and antioxidant activity of the aqueous propolis extract were lower than those of the ethanolic extract. Water and PBS did not differ in terms of phenolic content and antioxidant activity. Therefore, water can be preferred for various studies due to its greater accessibility, lower cost, and ease of preparation compared to PBS, depending on the specific requirements of the study.

The phenolic contents of propolis extracts in various solvents have been reported using different methods such as the TPC method (Alencar et al., 2007; Choi et al., 2006; Kumazawa et al., 2004) and the TFC method (Chang et al., 2002; Moreno et al., 2000; Silva et al., 2006), with antioxidant activity assessed using FRAP analysis (Kalogeropoulos et al., 2009; Mohammadzadeh et al., 2007; Moreira et al., 2008). The differences in the mechanisms used to measure polyphenol and flavonoid content and antioxidant activity across these methods can lead to minor variations in the results (Mello & Hubinger, 2012).

Given that the results of each method differ, the measured antioxidant activity, and the polyphenol and flavonoid contents of the extracts showed positive correlations. This indicates a direct relationship between antioxidant activity and the polyphenol and flavonoid contents in each extract.

Based on this information, the differences in phenolic content and antioxidant activity between propolis from Türkiye and other regions may be attributed to various factors, such as country, region, plant source, season, bee race, solvent used for extraction, and temperature. Additionally, no significant changes in phenolic content and antioxidant activity were observed in propolis samples from the Caucasian bee race within its gene region.

Although this research mainly investigated the effects of various solvents and temperature on Caucasian bee propolis obtained from different locations, it may shed light on future studies that require more detailed examination. For example, investigating how specific bee races, such as the Caucasian honey bee, interact with their local ecosystems and utilize different plant resources to collect propolis can provide important insights into the complex relationship between bee behavior, propolis composition, and ecosystem dynamics. In addition, the findings of the study are important for beekeeping practices and sustainability due to the important role of bee products, including propolis, in human health and well‐being. In the light of this information, beekeepers can realize more conscious and sustainable beekeeping activities by understanding how parameters such as extraction solvent and temperature selection affect the quality of propolis and other bee products. In addition, as a result of the analyses to be made with propolis and other bee products such as honey, pollen, and royal jelly of different bee races, it is possible to compare the bioactivity, composition, and extraction variables among these natural products. In this way, it can promote a comprehensive understanding of the various bee products produced by different bee races and their potential contributions to human health.

## 5 CONCLUSIONS

The phenolic content and antioxidant activities of propolis samples in Türkiye and other countries exhibit considerable variation. This variability is attributed to differences in phenolic content and antioxidant activity influenced by factors, such as country, region, plant source, season, climate, bee race, the solvent used for extraction, and temperature. Consequently, the differences in phenolic content and antioxidant activities of propolis samples from the Caucasian bee race's native region are primarily due to the solvent and temperature used during extraction, rather than differences within the bee race itself across locations. This pioneering study on Caucasian bee propolis suggests the potential for further research using propolis samples from different bee races in their native regions, thereby expanding the scope of such investigations.

## AUTHOR CONTRIBUTIONS

**Tuğba Nigar Bozkuş:** Conceptualization; formal analysis; funding acquisition; investigation; methodology; project administration; supervision; writing – original draft; writing – review and editing.

## FUNDING INFORMATION

This study was supported by Artvin Çoruh University Scientific Research Projects Coordinators (AÇÜ BAP), Türkiye under Grant No. 2021.M90.02.01.

## CONFLICT OF INTEREST STATEMENT

The author declares that there is no conflict of interest.

## Data Availability

The data that support the findings of this study are available from the corresponding author upon reasonable request.
